# Croton Oil in Vævus

**Published:** 1879-09

**Authors:** 


Croton Oil in Nævus.—I have had occassion to treat a case of nævus recently, and, as the treatment was unique as well as successful, I report it for the benefit of whom it may concern. The tumor was situated in centre of the left cheek, and in size was about as large as a dime. I procured a cork the size of the tumor, into which I inserted several fine needles, letting the points project one-eighth of an inch. I then immersed the points of the needles in pure croton oil, and plunged them into the tumor. A little swelling followed, and several vesicles formed soon after. The second day a crust formed over the whole tumor. This was repeated three times, at intervals of five days, and no other treatment was required. —*Mich. Med. News.*

